# Bone histology of Neogene angulate tortoises (Testudines: Testudinidae) from South Africa: palaeobiological and skeletochronological implications

**DOI:** 10.1098/rsos.230064

**Published:** 2023-03-08

**Authors:** Mohd Shafi Bhat, Anusuya Chinsamy, John Parkington

**Affiliations:** ^1^ Department of Biological Sciences, University of Cape Town, Private Bag X3, Rhodes Gift 7701, South Africa; ^2^ Department of Archaeology, University of Cape Town, Private Bag X3, Rhodes Gift 7701, South Africa

**Keywords:** bone microstructures, skeletochronology, Langebaanweg, Pliocene, tortoise, Western Cape

## Abstract

Here we examine the tibial microstructure of modern and fossil angulate tortoises to assess the histology and growth from the late Miocene–early Pliocene, Pleistocene through to modern forms. The cross-sections of all the tibiae sampled revealed highly vascularized, uninterrupted, fibrolamellar bone tissue during early ontogeny, which suggests that early growth was fast. However, later in ontogeny, growth was slower, as indicated by the deposition of parallel-fibred bone tissue in the outer cortex, and even ceased periodically, as indicated by lines of arrested growth. Comparative analyses of the growth rates of the tortoises from different time periods showed that the tortoises from the late Miocene–early Pliocene Langebaanweg locality and from Diepkloof Rock Shelter had relatively slower growth rates under less optimal growth conditions. Additionally, these prehistoric specimens show extensive remodelling, and several generations of secondary osteons further suggest functional and/or metabolic stresses on the skeleton. Palaeoenvironmental reconstructions suggest that it was mostly cooler and drier with seasonal fluctuations in late Miocene–early Pliocene, and it is likely that *Chersina* responded to these conditions by having a lower rate of growth as compared with their modern counterparts, which thrive in the current prevailing more favourable Mediterranean type of climate.

## 1. Introduction

Testudinidae [1] is an extant family of Testudinoidea that radiated from the paraphyletic basal group Lindholmemydidae [2–4]. The fossil record shows that the family first appeared in Asia during the Cretaceous–Palaeocene [5], and then colonized Europe, North America and Africa [6]. Africa has an exceptional chelonian diversity with several native species that date back to 35.5 Ma to the late Eocene [7–9]. Eleven of the 16 existing testudinid genera are found on the African continent, of which, six genera with 13 species live in South Africa [8,9]. Among the latter is the monotypic genus *Chersina* [10], known popularly as the angulate or bowsprit tortoise [11], which is endemic to South Africa and Namibia [12,13]. *Chersina angulata* is a medium-sized (carapace length = 350 mm) sexually dimorphic species, with males larger than the females [14], whose remains have been found in the early Miocene (Arrisdrift, Orange River, SW Africa; [15]) to Holocene [16,17], though a few studies suggest that the genus *Chersina* diverged from its sister group *Chersobius* during the mid-Oligocene (*ca* 30 Ma; [8]). Although the fossil record shows that *Chersina* was present at the Langebaanweg locality, in the southwestern Cape from the late Miocene to early Pliocene (*ca* 5.15 Ma; [6,15,18]), the extent of its distribution and the age of the taxon appears to be much older [19,20]. Angulate tortoises are primarily terrestrial and can inhabit a wide range of habitats and climatic zones [21,22], and they provide valuable information about ecosystem functions, such as seed dispersal and burrows [23,24]. Their remains are exclusively reported from archaeological or historical sites in South Africa [16,17] and it is likely that they were exploited by humans as a source of meat (chelonophagy *sensu* [25]) and for their shells [17,26]. In South Africa, their remains are primarily found as isolated shells and limb bones, as well as partial and articulated skeletons.

Long bone histology has long been used to infer growth dynamics and life history of extant and extinct vertebrates (e.g. [27–38], and has provided much insight into their age, bone growth rates, biomechanical constraints and sexual and skeletal maturity [27–33,39–51]. Although several histological studies have focused on growth dynamics of either extant or extinct taxa, few studies have investigated a single species over a long period of its evolutionary history (e.g. [52]). Moreover, although histological studies on chelonian limb bones and/or shell bones have been used to determine skeletochronology and reconstruct growth rates of the testudines [48,49,53–62], histological data from a single species, involving both modern and fossilized individuals is non-existent. Here, we fill this gap by investigating the bone histology of *Chersina angulata* from the Miocene to present, to assess whether their growth dynamics and life-history patterns changed over the past 5 million years.

## 2. Material and methods

In the present study, we analysed 49 tibiae of *Chersina angulata* (table 1). This study focused on tibiae, since an earlier study exploring the skeletochronology of *Chersina* showed that this element most reliably recorded the life history of the animal [48], and it also preserved histological features despite being subjected to damage caused by wildfires [49]. Specimens were recovered from the Pliocene palaeontological site at Langebaanweg (LBW), the Diepkloof Rock Shelter (DRS, which includes the provincial heritage site of Intermediate and Late Howiesons Poort occupation), the late Holocene locality at Dunefield Midden (DFM), as well as modern sites in Worcester and Cederberg. From each locality, we sampled different-sized bones, so that we could apply skeletochronology to deduce their ontogenetic age.

**Table 1.** Estimated length, cross-sectional diameter, relative bone wall thickness (RBT) and carapace length of studied tibiae.

registration number	TL (mm)	PW_L_ (mm)	PWs (mm)	MW_L_ (mm)	MWs (mm)	DW_L_ (mm)	DWs (mm)	RBT (%)	LAGs	CL (cm)
modern angulates (Cederberg)										
Ad-01	28.62	9.73	6.6	2.98	2.54	6.08	4.68	46	08	23
Ced-3 (R)	25.52	7.09	5.77	2.38	1.96	4.74	2.75	44.37	08	—
A64	24.36	6.67	4.79	2.5	1.68	4.14	3.59	40	07	—
Ced-4	24.10	6.88	5.54	2.59	1.84	4.78	3.56	35.5	07	—
Ced-2	24.70	8.25	6.0	2.71	2.41	4.97	3.84	43	07	—
A65	21.90	5.85	4.66	2.33	1.61	3.85	2.73	36	07	—
Ced-1	13.54	3.59	2.75	1.54	1.13	2.52	1.99	29	05	—
Onderplaas, Worcester (O/W)										
O/W-15/76 (M)	25.30	7.40	5.90	2.20	1.90	4.54	3.39	40.12	08	17
O/W-15/74 (M)	24.85	7.15	5.34	2.65	1.95	4.78	3.76	43.51	07	16
O/W-15/72 (M)	24.57	7.18	5.40	2.25	1.80	4.28	3.01	49.03	07	16
O/W-15/75 (M)	25.47	7.41	5.33	2.95	2.19	4.74	3.97	45.63	10	15.5
O/W-15/86 (M)	24.25	6.56	5.48	2.86	2.41	4.19	3.05	43.11	15	15.5
O/W-15/87 (M)	24.68	6.69	5.11	2.16	1.86	4.07	2.30	46.16	10	15.5
O/W-15/70 (M)	19.00	6.10	4.21	2.19	1.78	3.34	3.01	37.51	04	12.5
O/W-15/47 (F)	22.67	6.02	4.56	2.25	1.84	4.12	2.21	43.99	07	12.5
O/W-15/54 (F)	20.13	5.54	4.03	2.12	1.48	3.33	2.72	—	—	12.5
O/W-15/94 (M)	14.29	3.77	3.01	1.43	1.10	2.78	2.03	39.92	03	08
Dunefield Midden (DFM)										
DFM-JAC-20(R)	23.14	5.91	4.78	2.2	1.71	3.99	3.61	42.35	09	—
DFM-JAC-24 (R)	23.21	5.95	5.19	2.38	2.22	4.21	3.60	37.90	09	—
DFM-JAC-40(R)	20.30	5.48	3.60	2.22	1.9	3.87	2.58	41.01	—	—
DFM-SYL-43	20.32	4.69	3.58	1.84	1.17	3.32	2.23	43.07	05	—
DFM-SYL-44(R)	23.47	5.96	5.21	2.20	1.66	3.97	3.06	45.38	08	—
DFM-FRA-62	23.77	6.50	5.37	2.50	1.9	4.40	3.47	41.13	09	—
DFM-FRA-81	21.72	6.31	5.34	2.54	1.74	4.02	3.33	40.86	07	—
DFM-FRA-82(R)	21.87	6.35	5.17	2.52	1.91	4.23	3.41	42.50	07	—
DFM-FRA-99	20.12	5.30	4.76	2.19	2.01	3.54	2.56	38.17	07	—
Diepkloof Rock Shelter (DRS)										
DRS-M7B-Eve	26.44	7.22	5.19	2.32	2.02	4.20	3.26	47.75	09	—
DRS-M6C-01-Eve	22.71	5.72	4.71	1.98	1.68	4.03	3.28	42.35	08	—
DRS-N6C-Fiona	26.26	7.10	5.50	2.39	2.30	4.64	3.94	38.13	09	—
DRS-N9A-1-Fiona	28.96	7.75	6.02	2.74	2.51	4.77	4.25	47.87	10	—
DRS-N9A-2-Fiona	27.85	8.08	5.52	2.85	2.20	4.97	3.85	41.04	10	—
DRS-M6B-Fiona*	21.85	6.15	4.69	2.3	1.81	4.10	3.25	—	—	—
DRS-E6-2-George*	27.23	6.97	5.33	2.45	2.16	4.74	3.55	—	—	—
DRS-E6-1-George*	24.66	6.36	3.86	2.40	2.01	4.37	3.25	—	—	—
DRS-H6-George	27.19	6.58	5.22	2.76	2.23	4.47	3.34	44.95	09	—
DRS-H7D-George	27.71	6.88	5.41	3.20	2.45	4.81	4.01	45.85	—	—
DRS-G6-2-George	21.85	5.47	4.53	2.15	1.87	3.45	2.82	43.61	07	—
DRS-F6-George*	25.16	5.11	3.97	2.30	2.00	3.55	3.27	—	—	—
DRS-H7A-George*	22.52	5.96	4.18	2.60	2.38	4.23	3.94	—	—	—
Langebaanweg Fossil Park										
SAM-PQL-72347	27.96	9.53	6.76	4.20	2.88	5.94	3.84	—	08	—
SAM-PQL-72348	33.60	11.63	8.94	5.36	3.71	7.8	6.52	41.11	09	—
SAM-PQL-72349	29.30	10.10	6.93	4.62	3.14	6.25	5.78	43.36	09	—
SAM-PQL-72350	33.40	12.60	9.47	6.3	4.81	8.32	5.91	—	07	—
SAM-PQL-72351	29.47	9.70	7.29	3.91	3.60	5.98	4.56	43.98	09	—
SAM-PQL-72352	27.64	10.23	7.45	4.38	3.14	6.47	5.32	45.83	11	—
SAM-PQL-72353	20.60	7.89	5.49	3.7	2.30	4.87	3.78	—	07	—
SAM-PQL-72354	29.54	10.6	7.93	4.32	3.23	6.36	4.78	44.06	16	—
SAM-PQL-72355	23.02	7.54	6.32	3.82	2.84	5.17	3.95	—	11	—
SAM-PQL-72356	30.00	11.8	8.23	5.44	3.90	7.99	5.34	44.06	20	—

Note: When added to the following abbreviations: the subscript suffix L indicates a long axis; S indicates a short axis. Ced, Cederberg; CL, Carapace length; DFM, Dunefield Midden; DRS, Diepkloof Rock Shelter; DW, distal width; LAGs, lines of arrested growth; MW, mid-shaft width; PW, proximal width; RBT, relative bone wall thickness; SAM, South African Museum; TL, total length. Asterisks indicate that section is partially or completely lost during preparation.

### 2.1. Langebaanweg (LBW)

The palaeontological site of Langebaanweg (LBW) is located approximately 105 km northwest of Cape Town in the Western Cape Province of South Africa [63]. In the second part of the twentieth century, fossil-bearing pits were discovered and excavated in Langebaanweg fossil park during phosphate mining at Baard's Quarry [63–67]. Most of the vertebrate remains were later recovered from the ‘E’ Quarry, where fossils form extensive bone beds within the Mio-Pliocene Varswater Formation [18,68,69]. Previous research on LBW has proposed the Varswater Formation is deposited under estuarine, marine and/or fluvial environments ([70] and reference therein).

### 2.2. Diepkloof Rock Shelter

The archaeological site at Diepkloof Rock Shelter (DRS) is located approximately 180 km north of Cape Town [71,72]. Along with the neighbouring shelter, it dominates a largely isolated outcrop of quartzitic sandstone and is situated approximately 120 m above the southern bank of the Verloren Vlei River [72]. The site is 14 km from the Atlantic coast and the nearby site of Elands Bay Cave [72] and was first excavated by Cedric Poggenpoel and John Parkington in 1973; however, rock paintings on the shelter walls were discovered in 1960 [71,73,74]. The Rock Shelter is a storehouse of both Middle Stone Age (MSA) and Later Stone Age (LSA) faunal assemblages [75], encompassing pre-Stillbay, Stillbay, Howiesons Poort (HP) and post-Howiesons Poort components [72,76–78]. Bones from the DRS were recovered from hearths and as such, the skeletal elements were heated/burned to various degrees, but this did not obstruct the histological features [49]. The DRS tortoise assemblages analysed by us from this site are radiometrically dated to MIS 3 (i.e. 45–65 ka; [76,77]).

### 2.3. Dunefield Midden (DFM)

Another archaeological site, Dunefield Midden (DFM) is a late pre-colonial campsite located 2 km north of the mouth of the Verloren Vlei river in Western Cape Province, South Africa, and displays a series of brief occupations between 900 and 600 years ago, a time when the Holocene sea level was beginning to drop to its current level [26]. The base of this site is marked by Holocene-aged dune cordon exposed south of the active Dunefield, and the site is sandwiched between upper aeolian dune sands and an underlying coarser sand/pebble matrix [26,79]. The latter was probably deposited by standing seawater during a high sea-level stand when the Atlantic washed over the beach into an adjacent basin [80,81].

### 2.4. Modern localities

Modern tortoise skeletons studied were collected post natural fires in the Cederberg (approx. 300 km north of Cape Town) and Onderplaas, Worcester (approx. 110 km from Cape Town).

### 2.5. Anatomical measurements

Prior to the preparation of the histological thin sections, all the bones were measured and photographed. Standard measurements such as total length, mid-shaft diameters, and widths of proximal and distal ends were measured using Mitutoyo digital calipers with 0.01 mm precision (table 1). Different views of the bones were photographed using a Canon Power Shot SX60 HS. Note that all the bones were associated with partial carapaces without crania hence the snout–vent length could not be measured. All the bones of DFM, DRS and modern sites are from the repository of the Department of Archaeology, University of Cape Town (UCT) and LBW specimens were obtained from Iziko South African Museums, Cape Town.

### 2.6. Thin sectioning

For studying tortoise bone histology, thin sections were generated using cutting and grinding techniques following [82]. Thin sections were prepared from mid-shaft regions (i.e. 50% total length of the bone) as these areas are not extensively remodelled/resorbed and represent best preservation of primary bone tissue [29,31,48,83,84]. Before preparing resin blocks, bones were cut at mid-shaft levels using Dremel precision tool. Each half of the diaphysis was then ground and polished manually using carborundum (silicon carbide) discs of various grit sizes (P400, P600, P800, P1200), and was later embedded in epoxy resin (EpoxAcast 690 and/or Struers Epofix; [31,82]). The embedded bones were thereafter further ground down to expose the original cut surface of the bone. The polished side of each specimen was then mounted onto a glass slide using epoxy resin. The embedded specimens were then sectioned and ground using a Struers Accutum-50 precision cutter and grinder. Later, all the sections were further ground and polished on sequentially finer grit paper using a rotating Imptech 30 DVT grinder/polisher. This was followed by a final polish on a lap wheel with a velvet cloth using aluminium oxide (Al_2_O_3_) solution. The final thickness of the sections was approximately 30–35 µm. All the thin sections of the bones were studied and photographed using digital compact cameras Canon Power Shot D10 and Nikon DS-Fi1 mounted on Nikon Eclipse E200 and Carl Zeiss Axio Lab A1 polarizing microscopes, respectively. Bone wall thickness measurements were made using NIS Elements Microscope Imaging Software (v. 3.22.14), and the relative bone thickness (RBT), that is, the mean thickness of the cortical bone wall divided by the mean diameter of the total bone [31,85], was calculated. All the LBW specimens were obtained from Iziko South African Museums, Cape Town. Permission to section these fossils was obtained from the South African Heritage Resources Agency (SAHRA: permit 3098). DRS, DFM and modern tortoise specimens used in the current study were provided by the Department of Archaeology, University of Cape Town. The preparation and analysis of the histological sections was carried out in the Palaeobiology Research Lab, Department of Biological Sciences, University of Cape Town. The resulting thin sections are housed in the comparative osteology collection of the Department of Biological Sciences, UCT and Iziko South African Museums, Cape Town. The histological nomenclature follows Francillon-Vieillot *et al*. [29] and Chinsamy-Turan [31].

### 2.7. Perimeter measurements of lines of arrested growth

Skeletochronology is a technique in which the age of an individual is determined by counting the number of lines of arrested growth (LAGs) within the skeletal tissue [29,31,55,83,86–91]. All extant vertebrates lay down growth marks annually unless they attain maximum or full growth within a year [39]. Not all growth marks, however, are annual. Some appear randomly or stacked together within the compacta, and special growth marks are deposited at the time of hatching in reptiles [39], and at the time of birth in mammals (neonatal line) (*sensu* [92]). Indeed, often certain bones in a single skeleton, such as the stylopodium (humerus, femur) and zeugopodium (fibula, tibia, radius andulna), retain better growth mark record than the bones of cervical vertebrae, and in some cases, variation is noticed at different ontogenetic levels [62]. However, Bhat *et al*. [48] reported that the tibia retains better growth mark record in angulate tortoises. Experimental analyses have demonstrated that a cycle of rapid growth is deposited during the favourable growing season, and it usually corresponds to spring and summer months or rainy season [31,39,42,43,55,93]. On the other hand, a cycle of slowed growth formed during the unfavourable season is referred to as an annulus, and it is often associated with a LAG, reflecting abrupt cessation of growth [31]. In some cases, when periodic interruption is abrupt, only a LAG is deposited [55]. Thus, assuming that the LAGs are annual, counting the number of these LAGs in skeleton tissues provides a reasonable estimate of the age of the individual [31,39,55]. However, bone remodelling (*sensu* [94]) and secondary reconstruction often obliterate the record of early LAGs [31,32,83,95–97], and in certain cases, bone growth is too fast to deposit these annual rings [98]. It is possible that the LAG count does not always correlate with the real age of the individual [99]. Although the preservation of LAGs is affected by various factors [83,100], their annual cyclicity is generally accepted for both extant and extinct vertebrates [31,37,39,43,55,83,91,100–102].

In addition to estimation of the age of individuals [29,31,55,83,86–91], skeletochronology has been used to determine the season of death of the tortoises recovered from archaeological sites [49]. However, such osteohistological studies can also demonstrate the response of individual animals to environmental influences and constraints within their lifetimes [48,101,102]. In order to assess the growth dynamics of individuals, the perimeters/circumferences of LAGs were measured. By plotting these measurements for every individual, it is also possible to determine if earlier LAGs have been erased due to remodelling [47,91,102–105]. Full mid-diaphyseal tibial cross-sections were used to measure the perimeters of the LAGs. The actual perimeter measurements were obtained using the image analysis program, NIS Elements Microscope Imaging Software (v. 3.22.14). In cases where the trace of the LAGs could not be followed around the bone section, they were extrapolated using the outer circumference of the bone [106]. The bone perimeter measurements were plotted to reconstruct the growth series of angulate tortoises using the methodology of Bybee *et al*. [103], Woodward *et al*. [104], Orlandi-Oliveras *et al*. [47] and Chinsamy and Warburton [102]. To visualize and compare the growth dynamics of the tortoises from the different localities (i.e. Cederberg, Oonderplaas, DFM, DRS, LBW), all the data were plotted on the same graph (table 2).

**Table 2.** Perimeter measurements (mm) of growth marks. Ced, Cederberg; DFM, Dunefield Midden; DRS, Diepkloof Rock Shelter; SAM, South African Museum. ‘L’ in brackets is used for left skeletal element and ‘R’ is for right whereas ‘F’ is used for female individual and ‘M’ means male.

registration number	1	2	3	4	5	6	7	8	9	10	11	12	13	14	15	16
Modern angulates (Cederberg)																
Ad-01 (L)	4.2	4.9	5.5	6.3	6.8	7.3	7.7	8	—	—	—	—	—	—	—	—
Ced-3 (R)	3.9	4.6	5.1	5.5	5.9	6.3	6.8	7.3								
A64 (L)	3.4	4.2	4.8	5.4	5.9	6.2	6.7	—	—	—	—	—	—	—	—	—
Ced-4 (L)	3.8	4.6	5.2	5.9	6.4	6.8	7.3	—	—	—	—	—	—	—	—	—
Ced-2 (L)	3.9	4.7	5.3	5.7	6.3	6.8	7.4	—	—	—	—	—	—	—	—	—
A65 (L)	2.6	3.5	4.3	4.8	5.4	5.8	6.3	—	—	—	—	—	—	—	—	—
Ced-1 (L)	1.4	1.6	1.8	2	2.1	—	—	—	—	—	—	—	—	—	—	—
average	3.2	3.9	4.5	5	5.5	6.6	7.1	8								
Onderplaas, Worcester (O/W)																
O/W-15/76 (M)	3.8	4.3	5.0	5.5	6.2	6.9	7.3	7.4	—	—	—	—	—	—	—	—
O/W-15/74 (M)	3.1	3.7	4.2	4.9	5.6	6.1	6.8	—	—	—	—	—	—	—	—	—
O/W-15/72 (M)	3.3	3.9	4.5	5.1	5.5	6.1	6.5	—	—	—	—	—		—	—	—
O/W-15/75 (M)	2.4	3.6	4.1	4.6	5.3	5.7	6.2	6.6	7.2	7.6	—	—	—	—	—	—
O/W-15/86 (M)	2.5	3.4	3.8	4.3	4.8	5.6	6.1	6.4	6.8	7.1	7.2	7.2	7.5	7.7	8.1	—
O/W-15/87 (M)	2.9	3.3	3.9	4.5	4.9	5.3	5.6	5.8	5.9	6.4	—	—	—	—	—	—
O/W-15/70 (M)	2.9	3.7	4.4	5.1	—	—	—	—	—	—	—	—	—	—	—	—
O/W-15/47 (F)	3.1	3.5	4.2	4.7	5.2	5.7	6.4	—	—	—	—	—	—	—	—	—
O/W-15/94 (M)	2.2	2.9	3.5	—	—	—	—	—	—	—	—	—	—	—	—	—
average	3	3.6	4.2	4.8	5.4	5.9	6.4	6.6	6.6	7	7.2	7.2	7.5	7.7	8.1	
Dunefield Midden (DFM)																
DFM-JAC-20 (R)	2.2	2.7	3.2	3.7	4.3	4.8	5.3	5.7	5.9	—	—	—	—	—	—	—
DFM-JAC-24 (R)	2.9	3.6	4.1	4.5	4.9	5.6	6.1	6.5	6.9	—	—	—	—	—	—	—
DFM-SYL-43 (L)	2.6	3.5	4.2	5.1	5.7	—	—	—	—	—	—	—	—	—	—	—
DFM-SYL-44 (R)	2.4	2.6	3.4	4.4	5.3	6.2	6.8	7.1	—	—	—	—	—	—	—	—
DFM-FRA-62 (L)	2.8	3.2	3.6	4.2	4.5	5.1	5.4	5.8	6.3	—	—	—	—	—	—	—
DFM-FRA-81 (L)	4.1	4.4	4.8	5.2	5.5	5.9	6.2	—	—	—	—	—	—	—	—	—
DFM-FRA-82 (R)	3.7	4.4	4.5	4.8	5.2	5.7	6.3	—	—	—	—	—	—	—	—	—
DFM-FRA-99 (L)	3.3	3.7	4.1	4.3	4.7	4.9	5.2	—	—	—	—	—	—	—	—	—
average	3	3.5	4	4.5	5	5.5	5.9	6.3	6.4							
Diepkloof Rock Shelter (DRS)																
DRS-M7B-Eve	2.8	3.3	3.7	4.2	4.8	5.5	6.6	7.3	7.7	—	—	—	—	—	—	—
DRS-M6C-01-Eve	4.9	5.3	5.9	6.6	6.8	7	—	—	—	—	—	—	—	—	—	—
DRS-N6C-Fiona	5.2	5.9	6.2	6.6	6.9	7.2	7.5	7.8	8.1	—	—	—	—	—	—	—
DRS-N9A-1-Fiona	3.1	3.6	4.5	5.4	6.1	6.8	7.2	7.5	7.9	8.4	—	—	—	—	—	
DRS-N9A-2-Fiona	4.7	5.2	5.8	6.3	6.6	6.8	7.2	7.5	7.7	7.9	—	—	—	—	—	—
DRS-H6-George	—	—	—	5.1	5.6	6.2	6.7	—	—	—	—	—	—	—	—	—
DRS-G6-2-George	2.6	3.5	4.2	4.7	5.2	5.6	5.9	—	—	—	—	—	—	—	—	—
average	3.9	4.5	5.1	5.6	6	6.4	6.9	7.5	7.9	8.2						
Langebaanweg Fossil Park																
SAM-PQL-72347	4.2	4.6	5.1	5.5	6.1	6.5	7	7.4	7.6	—	—	—	—	—	—	—
SAM-PQL-72348	4.1	4.5	4.9	5.2	5.6	6.1	6.6	7	7.3	—	—	—	—	—	—	—
SAM-PQL-72349	4.9	5.3	5.7	6.2	6.6	7.2	7.5	7.7	8.1	—	—	—	—	—	—	—
SAM-PQL-72350	4.3	5.2	5.5	5.9	6.4	6.8	7	—	—	—	—	—	—	—	—	—
SAM-PQL-72351	3.8	4.5	4.8	5.2	5.4	5.9	6.4	6.7	7.3	—	—	—	—	—	—	—
SAM-PQL-72352	6.2	6.9	7.3	7.5	8.1	8.4	8.8	9.3	9.6	10	10.7	—	—	—	—	—
SAM-PQL-72353	5.2	5.6	6.1	6.7	7.1	7.6	8.1	—	—	—	—	—	—	—	—	—
SAM-PQL-72354	3.9	4.2	4.4	4.6	4.9	5.1	5.4	5.8	6.1	6.3	6.5	6.6	6.8	7	7.2	7.4
SAM-PQL-72355	3.5	4.2	4.8	5.2	5.5	5.7	6.1	6.4	6.7	7	7.3	—	—	—	—	—
SAM-PQL-72356	5.1	6.0	6.9	7.9	8.5	9.2	9.9	10.2	10.4	11.1	11.5	11.8	12.3	12.8	13	13.5
average	4.5	5.1	5.6	6	6.4	6.9	7.3	7.6	7.9	8.6	9	9.2	9.6	9.9	10.1	10.5

## 3. Results

### 3.1. Histological characterization of the modern tortoises from the Cederberg

**Figure 1.**
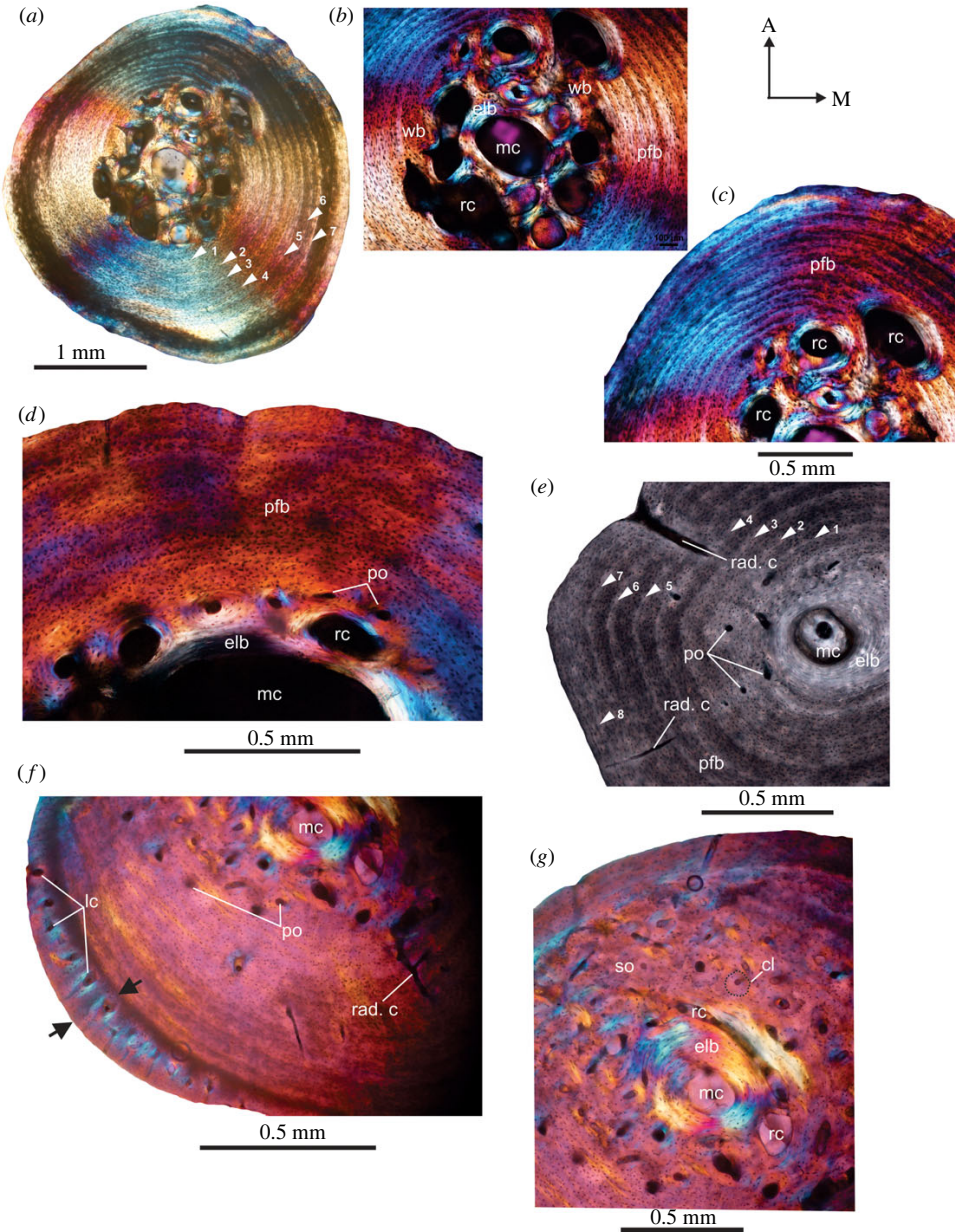
Transverse sections of modern tibiae from the Cederberg. (*a*) Overview of a diaphyseal cross-section of tibia (Ced-2; table 1) showing a central, open medullary cavity. Note the numerous enlarged resorption cavities in the perimedullary region. (*b*) Remnants of the early formed woven bone matrix. (*c*) Parallel-fibred bone tissue in the outer pericortical region. Note the orientation and density of osteocyte lacunae in the outer. (*d*) Transverse section of another individual (Ced-4; table 1) showing parallel-fibred bone tissue in the outer cortex. Note the density of osteocyte lacunae in the outer cortex. (*e*) Endosteal lamellar bone tissue enclosing medullary cavity in another specimen (Ced-3 (R); table 1). Note the primary osteons in the perimedullary region and the large radial canals that penetrate the compacta. (*f*) Development of a narrow richly vascularized band of tissue in the peripheral region (black arrows). Note the osteonal structures around these vascular canals. (*g*) Secondary reconstruction/remodelling in the mediolateral direction with numerous vascular canals. cl, cement line, elb, endosteal lamellar bone; mc, medullary cavity; pfb, parallel-fibred bone; po, primary osteon; rad.c, radial canals; rc, resorption cavity; so, secondary osteon; wb, woven bone. Numbers associated with arrow heads indicate LAGs in ascending order from the medullary region to the peripheral part of the cortex.

Histological analysis of all the tibial specimens from Cederberg reveal cross-sections with cortical regions composed of a thick compact bone walls (RBT = 29%–46%; table 1) which surrounds an open, small but clearly defined medullary cavity (figure 1*a*). At mid-shaft, the medullary cavity is centrally located (figure 1*a–e*), but in a few specimens, it is slightly displaced toward the lateral region of the cross-section (figure 1*f–g*). In most of the individuals (figure 1*a,d*), the medullary margins are irregular and highly resorptive, which contrasts with that of the largest individual in which the medullary cavity is small and sub-circular to circular, and there are fewer resorption cavities present (figure 1*g*). In several of the sections, the medullary cavities are surrounded by a thin layer of centripetally deposited lamellar bone (figure 1*b,d,e,g*). The inner and outer cortex is almost entirely composed of parallel-fibred tissue with fibres concentrically oriented with respect to bone axis (figure 1) and few vascular canals (figure 1*a–c*). However, in the perimedullary regions, small areas of primary woven bone tissue occur (figure 1*b*). The osteocyte lacunae are more abundant in the perimedullary regions, and they are less dense toward the periosteal margin (figure 1*d*). However, their density varies between individuals. In a few specimens, most of the cortex exhibits simple globular lacunae but they are flattened towards endosteal regions (figure 1*e*). In the largest individual (figure 1*f,g*), the cortex is thickest (RBT = 46%; table 1) and the specimen has an unusual localized periosteal growth of bony tissue in the lateral part of the bone wall (figure 1*f*). In this region, the tissue is also more vascularized, and the canals tend to have a radial organization (figure 1*f*). The medial margin also shows similar features, but to a lesser extent. Fibrolamellar bone tissue has a patchy distribution in the perimedullary regions (figure 1*f,g*) with longitudinally oriented primary osteons. Several LAGs occur throughout the cortex, and they interrupt the deposition of the parallel-fibred bone tissue (figure 1*a,e*); however, it is evident that in some individuals the earlier LAGs were erased due to remodelling (figure 1*g*). Eight LAGs are present in the largest individual (table 2), although most of the individuals have seven LAGs (figure 1*a,e*; table 2). A few isolated secondary osteons occur in the perimedullary region of some bones (figure 1*g*). Isolated radial canals are present in the cortex (figure 1*e,f*), and in a few instances they extend deep into the cortex. Several of the bones show secondary reconstruction, but in the largest individual (figure 1*g*), the oldest part of the cortex shows a few secondary osteons, several erosion cavities in the process of being infilled by endosteally formed lamellar bone, and very few open (unfilled) erosion cavities (figure 1*g*).

### 3.2. Histological characterization of modern tortoises from Onderplaas, Worcester

**Figure 2.**
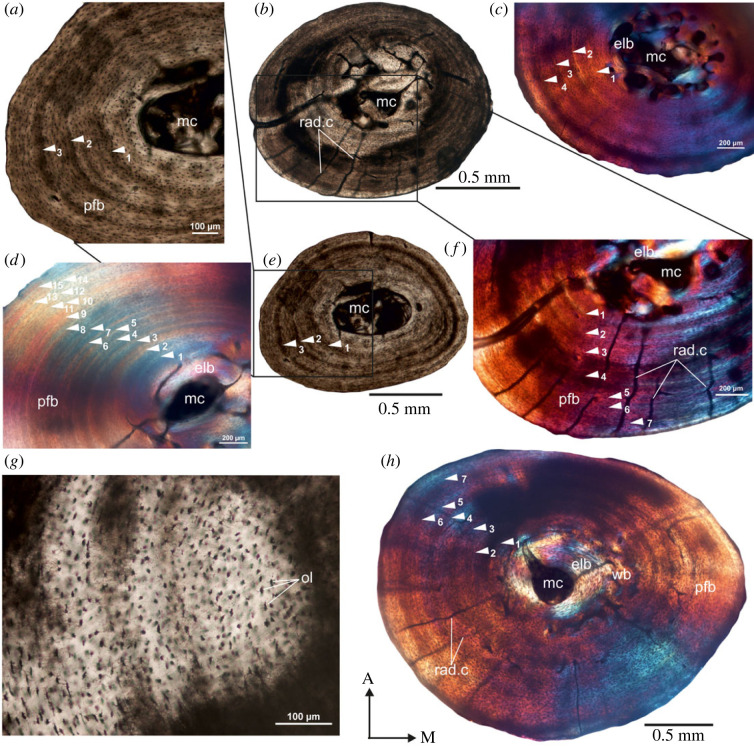
Transverse sections of modern tibiae from Onderplaas, Worcester, Western Cape. (*a,e*) Transverse view of a diaphyseal cross-section of tibia (O/W-15/94 (M); table 1) showing a large, open irregular shaped medullary cavity. (*b,f*) A diaphyseal cross-section of a tibia from a female (O/W-15/47 (F); table 1) showing large resorption cavities (*b*) and radial canals that extend into the compacta (*f*). (*c*) Parallel-fibred bone tissue in the outer pericortical region of another individual (O/W-15/70 (M); table 1) interrupted by several growth rings. (*d*) At least 15 LAGs are visible in the tibia of O/W-15/86 (M) (table 1). (*g*) Haphazard distribution of osteocyte lacunae in the compacta (O/W-15/87 (M); table 1). (*h*) Overview of a diaphyseal cross-section of tibia from a male, O/W-15/74 (M) (table 1) showing long radial canals that traverse the compacta. ol, osteocyte lacuna. For abbreviations, refer to legend of figure 1.

In all the bones from this locality, the medullary cavity is resorptive (figure 2*a–f*) and a ring of circumferentially formed lamellar bone tissue lines the medullary cavity (figure 2*b*). In general, the bone walls are composed of poorly vascularized parallel-fibred bone tissue (figure 2*a,c–e*) which enclose a central open medullary cavity (figure 2*a,d,e*). However, in a female tortoise (O/W-15/47; figure 2*b*) as well as in a few males (e.g. O/W-15/74; figure 2*h*), there are long radial canals that extend through the entire cortex. In a tibia of a female, the medullary cavity is small, and there are few enlarged erosion cavities present (figure 2*b*). Although, patches of fibrolamellar bone occur in some areas of the inner cortex (figure 2*h*), parallel-fibred bone tissue is the dominant component of all the cortices (figure 2). The entire cortices of the tibiae have high density of haphazardly arranged globular osteocyte lacunae; however, their density varies between individuals but remains homogeneous throughout the cross-section (figure 2*g*). They are flattened in the regions of endosteal bone tissues (figure 2*h*). Primary and secondary osteons are generally scarce in the compacta; however, few large erosional cavities occur in the perimedullary regions (figure 2b,c). Although, in a few specimens, the earlier LAGs may have been obliterated due to remodelling (figure 2*b,c*), a maximum of 15 LAGs, periodically interrupting parallel-fibred bone tissue, are noted (figure 2*d*). A male individual with the largest carapace length of 17 cm has eight LAGs in the compacta (table 1), while another male individual with a carapace length of 15.5 cm records the highest number of LAGs (i.e. 15; figure 2*d*; table 1).

### 3.3. Histological characterization of late pre-colonial tortoises (Dunefield Midden)

**Figure 3.**
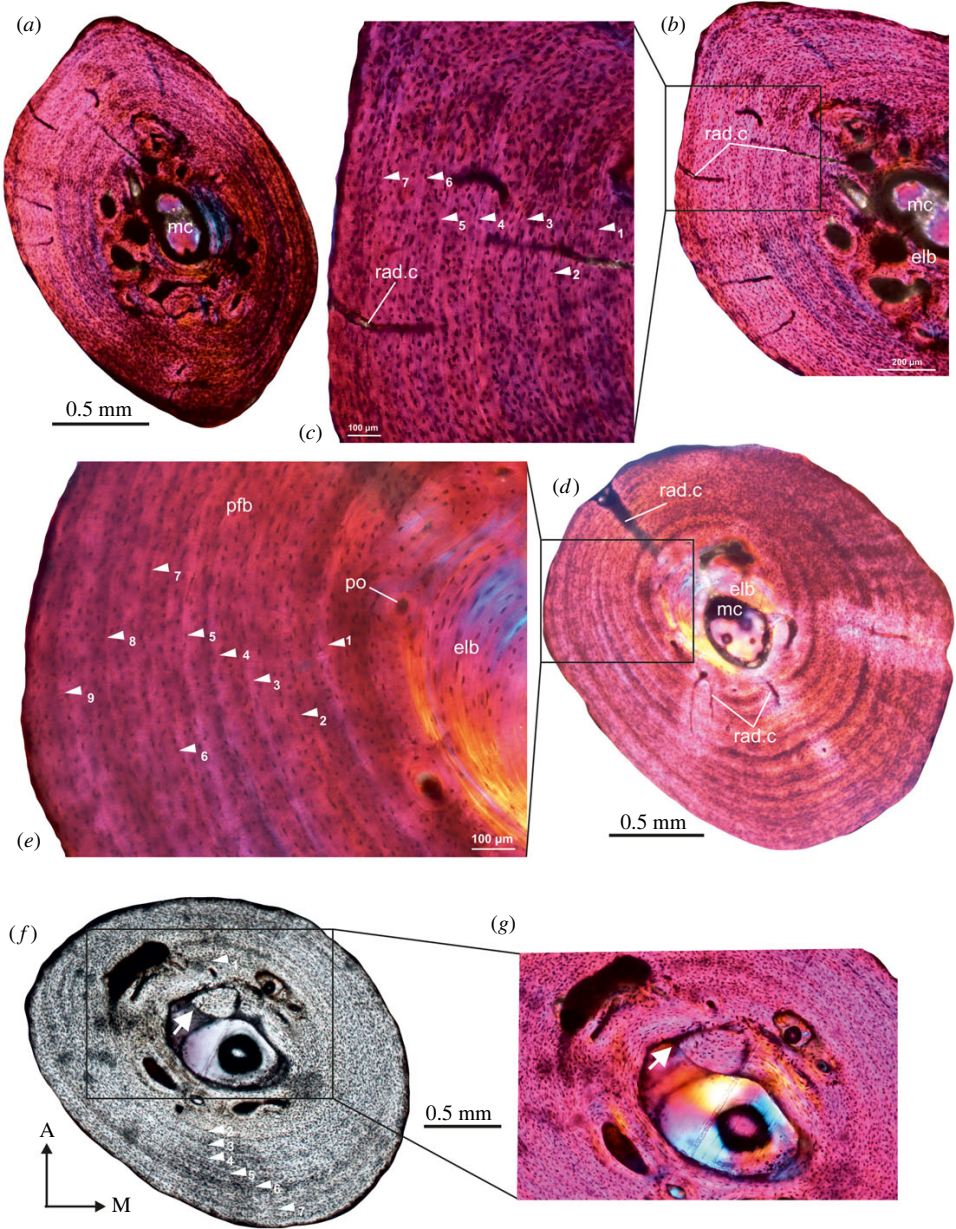
Transverse sections of tibiae from the late pre-colonial Dunefield Midden. (*a*) Overview of a diaphyseal cross-section of tibia (DFM-FRA-82(R); table 1) showing a central, open medullary cavity. (*b,c*) Parallel-fibred bone tissue interrupted by LAGs in the outer pericortical region (*b*) and haphazard distribution of osteocyte lacunae in the compacta (*c*). (*d*) Overview of a diaphyseal cross-section of DFM-FRA-62 (table 1) showing radial canals penetrating the compacta. (*e*) Density of osteocyte lacunae in higher magnification. (*f*) Poorly vascularized outer cortex with parallel-fibred bone tissue. (*g*) Large resorption cavities around the medullary region. Note: resorption in the endosteal region (white arrow). For abbreviations, refer to legend of figure 1.

The bones of the late pre-colonial tortoises have similar histological characteristics to the modern tortoises; however, they are comparatively more poorly vascularized (figure 3). The medullary cavity in these bones is sub-circular in outline (figure 3*a,d,f*) and is surrounded by a thin layer of endosteal lamellar bone (figure 3*e*). The thickness of the cortex varies around the bone cross-section (figure 3*a,d,f*), often with the medial side having the thicker compacta. The cortex of the bones is predominantly composed of parallel-fibred tissue, although there are a few enlarged erosion cavities concentrically arranged in the perimedullary region (figure 3*a,b,g*). There is a distinctive lack of secondary osteons throughout the compacta, although occasionally radial vascular canals traverse the bone wall (figure 3*a–c*). The osteocyte lacunae are abundant with a globular shape (figure 3*c*), preferentially arranged parallel to one another, whereas those in the endosteal lamellar bone appear to be more flattened (figure 3*e*). Several LAGs are evident in the cortex (figure 3*c–f*; table 1).

### 3.4. Histological characterization of intermediate to late Howiesons Poort tortoises (Diepkloof Rock Shelter)

**Figure 4.**
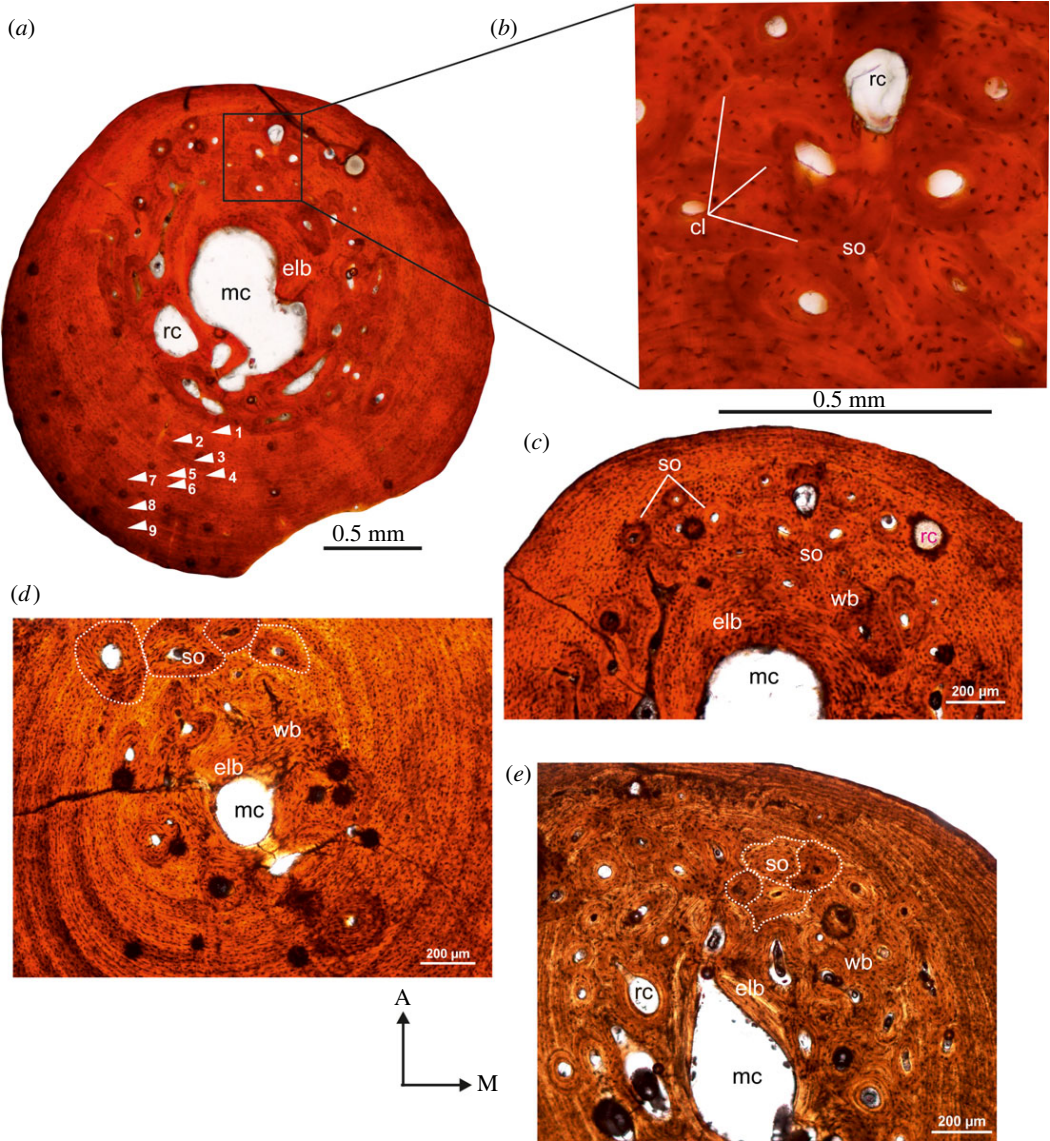
Transverse sections of tibiae from Diepkloof Rock Shelter. Note the distinctive orange colour in (*a*) and (*b*) is due to burning of the bones [49]. (*a*) Overview of a diaphyseal cross-section of tibia (DRS-N6C-Fiona; table 1) showing large, irregular, open medullary cavity. (*b*) Secondary reconstruction and several generations of secondary osteons arranged in haphazard pattern. (*c*) Transverse section of tibia (DRS-N6C-Fiona) under cross-polarized light with lambda compensator showing density of osteocyte lacunae in the outer cortex and long radial canals and several resorption cavities (rc). (Note there is no change in the colour of the bone after using filters, figure 4*c,e*). (*d*) Perimedullary region of the cortex of specimen DRS-N9A-1-Fiona (table 1) showing the density and distribution of secondary osteons in the perimedullary region. (*e*) DRS-N9A-2-Fiona (table 1), secondary reconstruction in the perimedullary region and several generations of secondary osteons arranged in haphazard pattern. For abbreviations, refer to legend of figure 1.

The thin sections revealed a thick cortical wall of compact bone with an open and discrete medullary cavity (figure 4). The peripheral margin is smooth, whereas the medullary cavity is resorptive, oval in shape and narrower mediolaterally (figure 4*a–e*). The outer cortex is predominantly composed of parallel-fibred bone (figure 4*a*), but parts of the compacta are more woven textured with numerous primary and secondary osteons (figure 4*b–e*). The cross-sectional histology reveals that there was a distinctive drift towards the posterior region of the cross-section, and this results in a distinctive asymmetrical distribution of the bone tissues (figure 4*a*). In general, parallel-fibred bone increases its thickness throughout the ontogeny (figure 4*a,c*), whereas the woven bone region appears to be early formed and less dominant in the cortex (figure 4*d*). Although secondary reconstruction is mostly restricted to the perimedullary region, in a few specimens, it extends mediolaterally, right to the periosteal margin (figure 4*e*). Several LAGs interrupt the deposition of the parallel-fibred bone tissue (figure 4*a*), and it is likely that LAGs formed during earlier stages of ontogeny may have been erased by remodelling (figure 4*e*). Ten LAGs were counted in two specimens, whereas the smallest specimen in the sample has seven growth marks (table 1).

### 3.5. Histological characterization of early Pliocene tortoises from Langebaanweg

**Figure 5.**
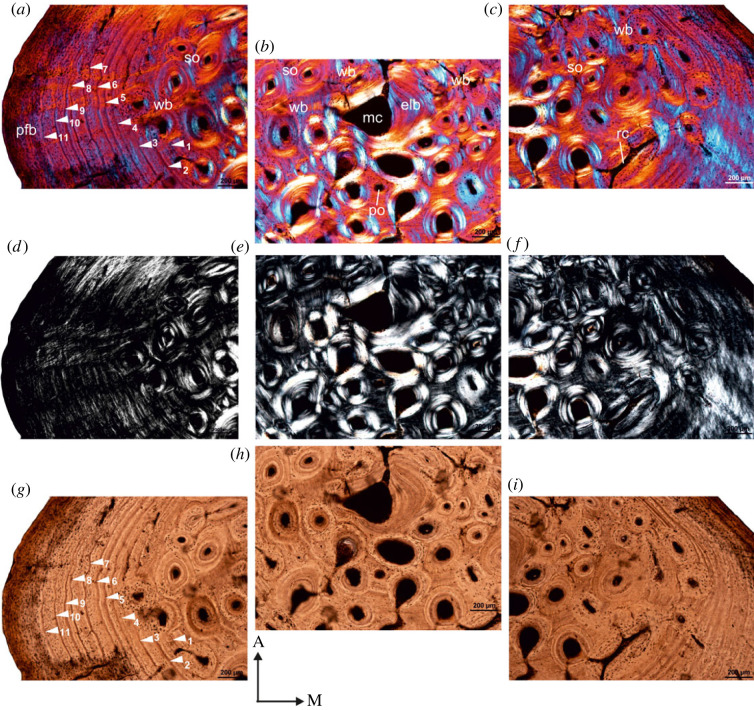
Transverse sections of tibia (SAM-PQL-72352; table 1) from the Pliocene locality, Langebaanweg, West Coast of South Africa. (*a,d,g*) Low-magnification images showing the cortical bone which is composed of parallel-fibred bone tissue interrupted by LAGs in the outer region and fibrolamellar bone in the perimedullary region. (*b,e,g*) Secondary remodelling in the perimedullary region and several generations of secondary osteons arranged in haphazard pattern. (*c,f,i*) Extension of secondary remodelling in the cortex and continued deposition of fibrolamellar bone, particularly in the lateral parts of the compacta, resulting in the formation of asymmetrical distribution of woven tissue. For abbreviations, refer to legend of figure 1.

**Figure 6.**
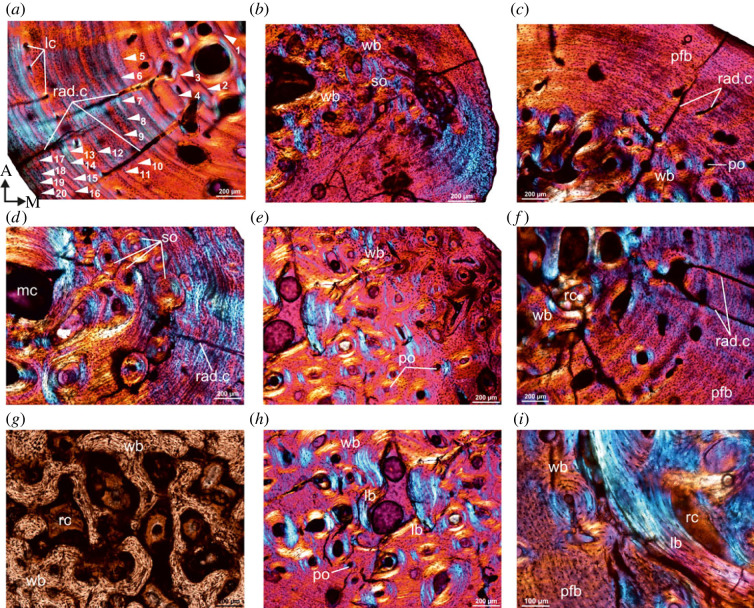
Transverse sections of tibiae from the Pliocene locality, Langebaanweg, West Coast of South Africa. (*a*) A low-magnification view of the cortical bone of tibia (SAM-PQL-72356; table 1) showing outer cortex of parallel-fibred bone tissue interrupted by 20 LAGs. Note: long radial canals that extend through almost the entire compacta. (*b*) High density of woven matrix in the perimedullary region of SAM-PQL-72349 (table 1). (*c*) Outer cortex of poorly vascularized parallel-fibred bone tissue with isolated long radial canals in the compact of SAM-PQL-72348 (table 1). (*d*) Secondary remodelling in the perimedullary region with several generations of secondary osteons (white arrows). (*e*) Extensive secondary remodelling in the cortex. Note: continued deposition of fibrolamellar bone, particularly in the lateral parts of the compacta, results in the formation of asymmetrical distribution of woven tissue. (*f,g*) Large resorption cavities in the perimedullary region. (*h,i*) Infilling of medullary cavity by endosteally formed lamellar bone. For abbreviations, refer to legend of figure 1.

A distinctive feature of the Pliocene bones is that the inner medullary regions are heavily remodelled (figures 5 and 6). The medullary cavity is remarkably small in these tibiae, and is often surrounded by a thin layer of endosteal lamellar bone tissue (figures 5*b* and 6*h,i*), but in a few specimens, the entire medullary region is infilled with secondary remodelled bone, and in some cases trabecular bone (figure 6*g*). The transition from the medullary region to the outer cortex is indiscrete. Instead, a large distinctive transitional region of compacted coarse cancellous bone occurs between the inner and the outer cortex (figures 5*c* and 6*e–h*). In a few specimens, compacted coarse cancellous bone occurs and is restricted to the medial region of the inner cortex. The primary cortex is made up of woven bone tissue richly vascularized by large, primary osteons, which are often eroded to form enlarged erosion cavities, some of which are lined by lamellar bone tissue. Several generations of secondary osteons and enlarged cavities are present in the inner regions of the compacta, although in some tibiae they reach the medial peripheral margin. In addition, their density is much higher in areas where the primary cortex grades into the coarse cancellous bone (figure 6*d,e*). Overall, the outer cortex is dominated by parallel-fibred bone tissue with globular osteocyte lacunae arranged parallel to the peripheral margin (figures 5*a* and 6*a–c*). In a few specimens, long radial canals penetrate almost the entire outer compacta (figure 6*a,c,f*), where there is a marked decrease in the density of vascular canals. A maximum of 20 LAGs were counted in the outer cortex, and given the extensive secondary reconstruction, it is likely that earlier formed LAGs were removed (figure 6*a*). It may be noted that the largest specimen (SAM-PQL-72348, 33.6 mm in total length, table 1) has nine LAGs in the compacta compared.

## 4. Discussion

### 4.1. Histological variations and palaeobiology of *C. angulata*

The tibiae from DRS and LBW showed substantial variation in their growth dynamics through ontogeny. They showed heavily remodelled inner medullary regions, predominant fibrolamellar tissue in the inner cortex, several generations of secondary osteons, enlarged resorption cavities and a higher number of LAGs in the compacta. On the other hand, the overall growth of tortoises from the archaeological site Dunefield Midden (DFM) along with the modern specimens from Cederberg and Onderplaas showed much slower growth patterns, as illustrated by the predominance of slowly forming parallel-fibred bone tissue with LAGs in the outer cortex. LAGs are also present in the outer cortex of DRS and LBW tortoises, although they exhibited different bone tissue types in their compacta. The occurrence of multiple LAGs in the cortex indicates a cyclical or flexible growth strategy [31,48,49,87–89,107]. Such growth dynamics are probably linked to prevailing environmental conditions (e.g. [93,101,102]).

Overall, the DRS and LBW tortoises show similar histological pattern to the tortoises from the 600–900-year-old DFM site, and the two modern sites (Cederberg and Onderplaas) (i.e. outer cortices of parallel-fibred bones surrounding inner woven bone), suggesting fast growth early in ontogeny followed by slow growth until the animals died (e.g. [31,48,49]). However, the tortoises from DRS and LBW continued to deposit fibrolamellar bone for a longer period, particularly in the lateral parts of the compacta, which results in the formation of asymmetrical distribution of woven tissue in the compacta. Bhat *et al*. [48] noted this unidirectional deposition of fibrolamellar bone tissue in propodials (femora and humeri) of modern tortoises and suggested the influence of differential functional constraints on the bone histology in these bones. These histological variations observed in the compacta are linked to changes in the bone deposition rates and are commonly seen in vertebrates including chelids [60,84,108–110]. In most of the specimens from DRS and LBW, comparatively high vascularization levels occur, but in the outer more poorly vascularized part of the compacta, long radial canals are present. Generally, the higher degree of vascularization suggests higher osteogenetic rates [111] as they are the sites where nutrients are assimilated in the bone [43]. This also suggests large supply of blood vessels and high nutrient demands for the animal during the periods of rapid bone depositional rates [31]. In modern tortoises, in addition to simple longitudinal vascular canals, long radial canals were observed in the compacta of female individuals [48], and in this study, we found a female tortoise with an egg in its body cavity that also had an abundance of these long radial canals. Based on these findings, we cautiously propose that the fossil specimens from LBW which show large radial canals penetrating the compacta may also represent females; however, this warrants further testing on a larger sample.

During late stages of ontogeny, in the tortoises from Dunefield Midden (DFM) and the two modern sites (Cederberg and Onderplaas), there is a marked increase of the organization in the collagen fibres with the deposition of poorly vascularized parallel-fibred bone in the outer cortex. In South American long-necked chelid turtles, Pereyra *et al*. [60] identified two patterns of organization of the collagen fibres: fibres in one direction (concentric or longitudinal with respect to the main axis of the bone) and fibres in two directions (concentric and longitudinal with respect to the main axis of the bone, i.e. crossed parallel-fibred bone). However, we did not notice such variations in the orientation of collagen fibres in the parallel-fibred bone complex, although local variations in the density of osteocyte lacunae and vascularity through ontogeny was noted.

Another interesting finding is that the tortoise remains from archaeological site DRS and palaeontological site LBW showed intense secondary reconstruction [31,32,97] in the perimedullary regions, but extending to the peripheral margins in some instances. However, as compared with individuals from LBW, the secondary osteons are not as extensively developed in DRS tortoises except for one individual (figure 4*e*; [49]). Generally, a high degree of bone remodelling increases with age [29,31,94,112,113] and may be linked to a number of factors such as physiological and biomechanical stresses [31,94,95,113–116]. In addition, secondary reconstruction is also linked to the formation of bone tuberosities and muscle attachment sites [94,117,118]. Recently Montoya-Sanhueza *et al*. [119] suggested that bone remodelling in naked mole-rats may be associated with activities of high metabolism (e.g. relocation of diaphyseal structures and reproduction). However, this may not be the case in tortoises, as the specimens which came from females lack secondary osteons but have dense vascularity in the perimedullary region and long radial canals traversing the outer poorly vascularized cortex. In addition, in a few specimens, irrespective of their sex, there appears to be a concentration of simple longitudinal and radial canals in the vicinity of areas of muscle attachments.

In one individual (Ad-01, table 1), in the mid-shaft region, the bone surface has bleb-like protrusion. The transverse section of the element revealed that this region showed a distinctive outwardly directed bony growth with high vascularity (figure 1*f*). Based on the histological observations, we propose that this tissue represents a periosteal reactive growth. Similar pathological conditions were observed in the ribs of *Proneusticosaurus silesiacus* and have been attributed to tuberculosis-like respiratory infection [120]. Such reactive growth has been reported in therapsids [121], sauropodomorph dinosaurs [122] and extant and extinct phocid seals [123]. However, in our specimens, although similar osteological and histological features were noted, the pathology appears to be more localized, and may have been due to some unknown injury.

Overall, the bone microstructures of *C. angulata* (irrespective of the age of the sample) is comparable to that reported for the aquatic turtle *Podocnemis expansa* [55] and *Pelomedusa subrufa* as well as the tortoises *Homopus femoralis* [8,56], *Testudo hermanni*, *Testudo graeca*, *Astrochelys radiata*, *Geochelone carbonaria* [124] and *Stigmochelys pardalis* [125]. The predominance of parallel-fibred bone tissue in the pericortical region of the tibiae implies similar growth rate as in most testudines except for the leatherback sea turtle, *Dermochelys coriacea* [56], which has entirely spongious limb bones [126] as an adaptation for their pelagic lifestyle. However, Bhat *et al*. [48] reported on the occurrence of fibrolamellar bone tissue in the perimedullary to mid-cortical regions of several modern tortoises. In the current study, remnants of early formed fibrolamellar bone tissue is preserved around the endosteal margins in modern tortoises recovered from Onderplaas. However, tortoises recovered from DRS and LBW are distinctive in having thick bands of fibrolamellar bone tissue similar to that described in the juvenile leopard tortoise, *Stigmochelys pardalis* [125] and propodials of modern tortoises [48]. This contrasts with observations of the tortoises from the archaeological site, Dunefield Midden (DFM) where the entire cortex is composed of parallel-fibred bone. In most of the specimens, generally, vascularization decreases substantially towards the peripheral cortex, indicating a decrease in growth rate [43], and is similar to observations in *Stigmochelys pardalis* [125], although in this case, simple canals are embedded in the parallel-fibred bone.

In the modern angulate tortoise, Bhat *et al*. [48] reported on the deposition of the bone tissue toward posterior margin and resorption in the anterior side of diaphyseal section (cortical drift; [94]), which resulted in cortical thickening toward the posterior side of the femora and humeri of *C. angulata*. Such histological features have also been noted in naked mole-rats, *Bathyergidae* [127] and in most of the specimens of *C. angulata* described in this study and suggest that the overall asymmetrical growth of the compacta might be related to the digging behaviour.

### 4.2. Skeletochronology

**Figure 7.**
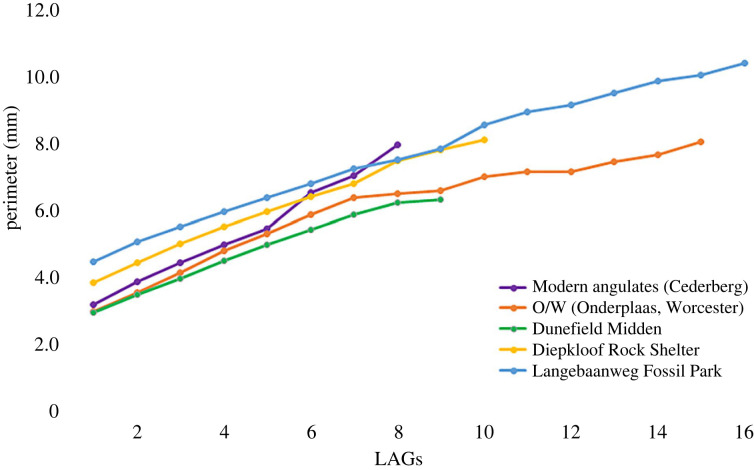
Growth curves obtained by plotting the perimeter of each LAG (mm) against the growth mark (i.e. age in years).

Branch [128] reported slow growth for the angulate tortoises with attainment of sexual maturity at about 10–12 years in the wild and 7 years in captivity. In a skeletochronological assessment of angulate tortoises, Bhat *et al*. [48] reported that an adult individual (snout–vent length approx. 230 mm) reached sexual maturity approximately at 6–8 years. In the current study, several distinct growth marks are observed in all the individuals and most of them are preserved in the outer cortex, which is dominated by parallel-fibred bone tissue. However, the number of growth marks preserved in the cortex is variable, with the most variations evident in specimens recovered from LBW. The specimen SAM-PQL-72356 (tibial length is 30 mm, table 1) has 20 growth rings visible in the cortex (figure 6*a*), but it must be noted that this reflects a minimum estimation of age [89,91]. It is interesting that the early growth marks in SAM-PQL-72356 are relatively wide, whereas they become more narrowly spaced in the outer cortex (figures 5*g* and 6*a*). It is evident that the 8th–9th growth mark distinctly shows a transition to narrow growth rings (figure 7). (Note that, although in some specimens there is some variation in the thickness of the zones (figure 2*d*), in general the spacing of the 8th–9th growth marks typically marks the transition to a slower pace of growth irrespective of the geological age.) Based on these findings, we propose that these tortoises reach sexual maturity in the 8th year [55,91,129,130]. It is worth noting that identification and interpretation of LAG spacing is highly complicated since it changes in cross-section due to the general architecture of the bone [55], and we believe that it is possible that the sexual maturity of angulate tortoise may be overestimated, as the number of young individuals in the dataset is unknown. However, it is evident from this study that most of the individuals in our sample have attained sexual maturity much earlier than previously proposed [128], and this further corroborates the findings of Bhat *et al*. [48,49].

### 4.3. Growth series and evolutionary implications

In extant and extinct vertebrates, growth dynamics is interpreted by analysing growth rates through ontogeny [83,131,132]. Perimeter measurements of the LAGs permit a better estimation of growth dynamics; however, it assumes that individual animals grow at the same rate and does not consider variations in growth rate through lifetime (see [102]). In the present study, LAG counts and their perimeters across the cross-section were calculated and plotted [31] to reconstruct the growth of tortoises from the different localities (figure 7; [83,102]). It may be noted that growth cycles are usually difficult to discern since many of the LAGs are closely spaced, and it is often difficult to differentiate between LAGs that are closely adjacent to one another. In addition, considering that not all LAGs may be preserved in the compacta, the age estimation based on LAG count is always the minimum age at the time of the animal's death [37,99]. However, we assume that it is unlikely that individuals were older than estimated, given that the partial traces of LAGs are visible in the remodelled regions of the cross-section and/or may be extrapolated throughout the cross-section (*sensu* [106]).

**Figure 8.**
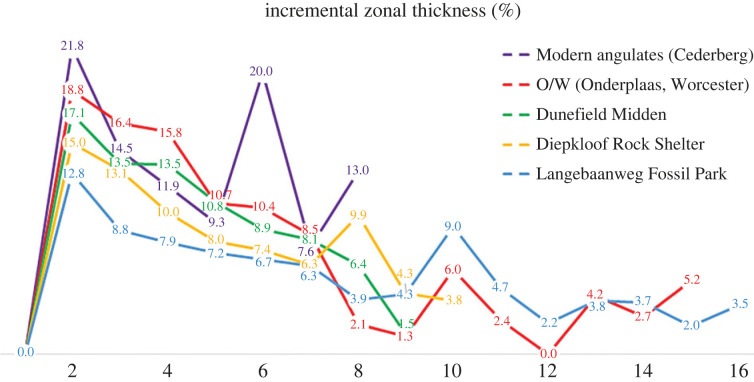
Graphical representation of incremental zonal thickness (in percentage) at each location.

The data retrieved for modern tortoise remains from Cederberg and Onderplaas follow similar growth trajectories early in ontogeny; however, Cederberg tortoises show a steeper growth rate later in ontogeny (figure 7), and it appears that they not only grow fast early in ontogeny but the zonal thickness between LAGs is much larger (figure 7) than the DRS and LBW tortoises. It is noteworthy that the average perimeter of LAG 1 at DRS corresponds to the same value of LAG 2 at LBW (table 2). Both reach the same value at LAG 9 and thereafter, the DRS tortoises grow at a slower rate than the LBW tortoises. Although all the tortoises grow faster during early stages of ontogeny, it is evident that the modern tortoises have the fastest rates of early growth as indicated by the zonal thickness (20–22%), whereas the tortoises from LBW have the smallest thickness (figure 8, table 2). Thus, despite the deposition of fibrolamellar bone tissue, the perimeter/LAG count measurements showed that the overall growth rate of the archaeological (DRS) and palaeontological (LBW) tortoises is slower when compared with the modern specimens. We suggest that, since climate was in a state of transition during the Pliocene (at Langebaanweg, see below, e.g. [133,134]) this might have influenced their growth rates. Curiously, it appears that the diameter of the bones of the LBW were the largest of all the bones samples (figure 7), which means that hatchling size of the tortoises was considerably larger than that of modern forms, with the smallest hatchling size recorded for the Dunefield Midden site. Another point worth noting is that although initially all the slopes of the growth curves (figure 7) are similar, for some reason, in the modern sample, after the sixth LAG there appears to be a spike in the growth rate (figures 7 and 8). We suggest that this may have been in response to extremely favourable conditions for growth (such as water and food availability). Note that similar spikes in growth during particular ‘seasons’ are also evident for the LBW, Diepkloof and Onderplaas samples, whereas the Dunefield and Onderplaas tortoises also show an occasional depression in growth (figures 7 and 8), which might reflect particular unfavourable seasons.

### 4.4. Environmental implications and habitat

The cooling effect from the Benguela current, as well as the latitudinal, seasonal movement of the South Atlantic high-pressure system, have a significant role on the present climate of southwest South Africa [134]. Summers are usually dry, hot and windy, whereas winters are cold and wet, resulting in a rich abundance of fynbos species with C3-dominated floral communities [134–137]. Previous research has linked tortoise mobility to resource availability, reproductive status and local environmental variables [24]. In South Africa, angulate tortoises are commonly found in Mediterranean-type climates [128] and prefer regions with low annual rainfall and mild to hot summers with temperatures ranging from 23 to 33°C [138]. However, inland populations are also found in areas with higher rainfall [14]. In the southern and western Cape, angulate tortoises are found on sandy, acidic, nutrient-poor soils with low shrub coastal fynbos vegetation composed of grasses [14]. However, they also occupy rocky areas [14,138]. Generally, angulate tortoises are herbivorous, feeding on annuals, grasses, flowers and succulents [139] and have also been known to eat snails, insects, moss, mushrooms and the occasional animal faeces [14]. As tortoises thrive in environments which have predominantly C3 canopy with few species preferring the intake of C4 plants (e.g. *Aldabrachelys gigantea*; *Geochelone* *grandidieri*; [140]), angulates in particular are found in high densities in such regions [14,141]. This may be the reason for rich diversity of testudines in South Africa, where more than six genera with 13 species inhabit the rich floristic regions of Western Cape [142].

The climate at the time of occupation at DFM is assumed to have been relatively similar to the present with hot, dry summers and cool, moderately wet winters [143]. Our histological results of the modern tortoises (Cederberg and Onderplaas) and archaeological remains of DFM suggest favourable environment conditions for the growth of the tortoises.

The Diepkloof Rock Shelter document the occupations from the MSA, but some LSA components are also recorded in the form of superficial archaeological layers, pits excavated into the MSA deposits, and paintings on the wall of the shelter [72,78]. At present, the surrounding vegetation around the Diepkloof Rock Shelter consists of semi-arid shrublands, adapted to long, hot summers and brief, cold, wet winters [75]. The climate at the time of occupations reflects a shift toward drier conditions, as evidenced by the dominance of Afromontane forest and thicket species during Still Bay occupations and more thicket and shrubland woody taxa during Howiesons Poort occupations [144,145]. Tortoise bones are abundant in the DRS sequence [75], and MSA specimens are much larger than LSA specimens, most likely because LSA human communities preyed more heavily on tortoises [75,146].

Dental mesowear and stable isotope studies of fossilized animals at the Langebaanweg fossil site show that during the early Pliocene (i.e. *ca* 5.15 Ma), the local environment at LBW remained C3-dominated canopy [67,133,147]. It is also assumed that Langebaanweg at approximately 5 Ma years ago was a heavily forested region with little grass [67,133]. Early reconstructions of the palaeoenvironment of the LBW fossil site suggest that it was in a state of transition throughout the late Miocene and early Pliocene with woodlands/forests being gradually replaced by open grasslands [133,134]. However, the presence of a rich and diverse frog population at LBW indicates that the rainfall on the west coast was significantly higher in the early Pliocene, and the aridification had not yet begun in the west coast as seen today [147]. In addition, the data obtained from stable isotope analysis also suggest that rainfall was low in winter [133]. Both the DRS and LBW tortoises show more or less a similar growth pattern with the inner cortex dominated by fibrolamellar bone followed by the outer cortex of parallel-fibred bones tissue. However, the inner cortex of LBW tortoises is heavily remodelled as compared with the DRS tortoises. The overall growth rate is slow for LBW remains as compared with the DRS individuals. We propose that the tortoises from modern sites and DFM thrived under the more favourable climatic regime of dry, hot and windy summers with cold and wet winters. Contrarily, the subtropical climate during the LBW was feasible for C3-dominated canopy, but winters were cold and drier than the current climatic system of South Africa, similar to DRS site, which is probably responsible for the slower growth rates exhibited by the tortoises from Langebaanweg and DRS locality (figure 8).

## 5. Conclusion

The comparative analysis of skeletal elements of angulate tortoises recovered from the Pliocene palaeontological site at Langebaanweg (LBW), through several highly significant rock shelters, including provincial heritage site of Diepkloof Rock Shelter (DRS), to late pre-colonial site of the terminal Holocene at Dunefield Midden (DFM) and modern sites at Worcester and Cederberg provided relevant information about the life history of these tortoises over a considerable period of time. Our results revealed that these tortoises experienced cyclical growth dynamics with faster rates of bone deposition during early stages of ontogeny, and slower rates during later stages of ontogeny. Here for the first time we document an abundance of fast-growing fibrolamellar bone tissue and profuse secondary remodelling in the inner cortices of *C*. *angulata* bones recovered from several sites ranging from modern localities to the Pliocene. The presence of LAGs in the compacta, suggest that these tortoises were susceptible to environmental fluctuations [101,102]. We also deduced that sexual maturity probably occurred at 7–8 years of age. Finally, the study also revealed that the modern tortoises experience the fastest rates of growth, whereas the Pliocene LBW tortoises had the slowest growth rates, which may have been influenced by the unstable environmental conditions that prevailed at that time. The bone histology of a multiple number of specimens from different localities also shed light on the fossorial behaviour of the animals. Most of the specimens show asymmetrical distribution of the bone wall (i.e. more prominent deposition of bone in the posterior region, with resorption in the anterior region of the diaphyseal section, and is usually seen in fossorial animals [48,148–150]) resulting from their digging lifestyle [48,49,127,151].

## Data Availability

This article has no additional data.
